# Supplementary material for: The Non-JAZ TIFY Protein TIFY8 of Arabidopsis thaliana Interacts with the HD-ZIP III Transcription Factor REVOLUTA and Regulates Leaf Senescence
Source: Int J Mol Sci. 2023 Feb 4;24(4):3079. doi: 10.3390/ijms24043079 (PMC9967580; doi:10.3390/ijms24043079)
Supplement: Supplementary file 1 [file ijms-24-03079-s001.zip › ijms-2170636-Supplemental Figures.pdf]

International Journal of *Molecular Sciences*

MDPI

"The non-JAZ TIFY protein TIFY8 of *Arabidopsis thaliana* interacts with the HD-ZIP III transcription factor REVOLUTA and regulates leaf senescence."

Ana Gabriela Andrade Galan [1], Jasmin Doll [1], Svenja Corina Saile [1], Marieluise Wünsch [1], Edda von Roepenack-Lahaye [1], Laurens Pauwels [2,3], Alain Goossens [2,3], Justine Bresson [1] and Ulrike Zentgraf [1,*]

[1] Center for Plant Molecular Biology (ZMBP), University of Tübingen, Auf der Morgenstelle 32, 72076 Tübingen, Germany;

[2] Department of Plant Biotechnology and Bioinformatics, Ghent University, B-9052 Ghent, Belgium

[3] Center for Plant Systems Biology, VIB, B-9052 Ghent, Belgium

* Correspondence: ulrike.zentgraf@zmbp.uni-tuebingen.de

Supplemental Figures S1-S12

(a)

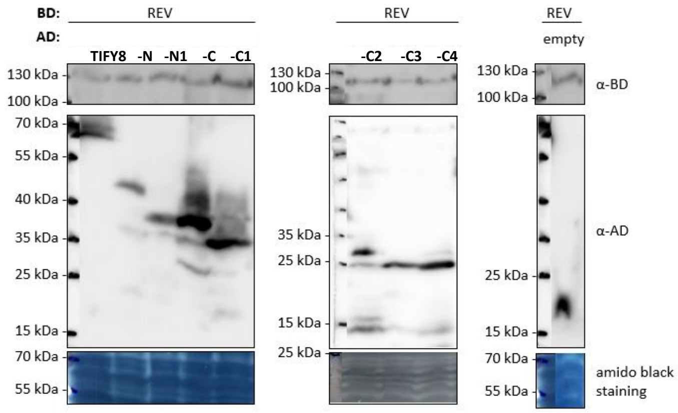

(b)

BD	AD	TDO	DDO/X/AbA	QDO	QDO/X	QDO/X/AbA
REV	TIFY8	83%	17%	83%	0%	17%
REV	TIFY8-N	20%	0%	20%	0%	0%
REV	TIFY8-N1	0%	0%	0%	0%	0%
REV	TIFY8-C	100%	20%	60%	100%	40%
REV	TIFY8-C1	80%	20%	0%	0%	0%
REV	TIFY8-C2	0%	0%	0%	0%	0%
REV	TIFY8-C3	0%	0%	0%	0%	0%
REV	TIFY8-C4	40%	20%	0%	0%	0%
REV	empty	0%	0%	0%	0%	0%

100%
83%
80%
60%
40%
25%
20%
17%
0%

Figure S1: *Yeast two-hybrid protein expression in yeast cells and heat map of positive results in independent mating experiments.*
(a) Western blot analyzed with anti-BD and anti-AD antibodies (α) show expression of BD-REV and AD-TIFY8 fusion proteins in diploid yeast cells. Protein loading is indicated by a section of the Amido Black Staining (A.B.S) BD-REV: 114 kDa, AD TIFY8:62 kDa, AD-TIFY8-N: 48 kDa, AD-TIFY8-N1: 43 kDa, AD-TIFY8-C: 44 kDa, AD-TIFY8-C1: 38 kDa, AD-TIFY8-C2: 33 kDA, AD-TIFY8-C3: 30 kDa, AD-TIFY8-C4: 30 kDa, AD-empty: 22 kDa. **(b)** Heatmap of positive interaction results of 3 to 7 independent mating experiments in % positive results.

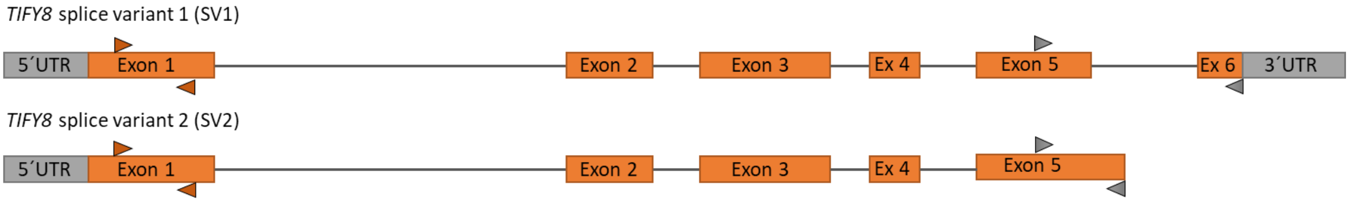

Figure S2: *Splicing variants of TIFY8.*
Two splicing variants of *TIFY8* differ in the 3´region in exon 5 and 6. Primer position used for qRT-PCR (orange) and splicing variant determination (grey) are indicated by the arrow heads).

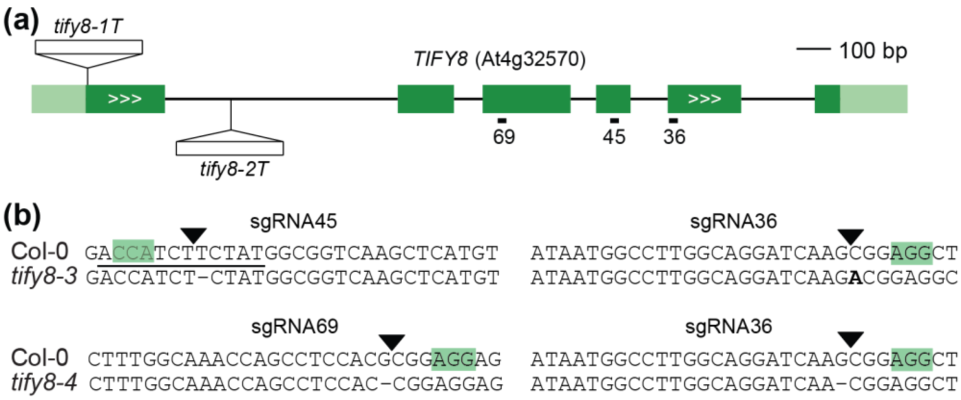

Figure S3: *Generation of tify8 CRISPR/Cas9 lines*.
(a) Genomic structure of Arabidopsis *TIFY8* and location of the T-DNA inserts and sgRNAs. Dark green boxes designate exons; light green boxes, UTRs; solid lines, introns; white arrows gene orientation. sgRNA numbers are arbitrary identifiers. Locations of the *tify8-1T* and *tify8-2T* T-DNA are indicated. **(b)** Genotypes of homozygous *tify8-3* and *tify8*-4 mutants, generated by combining sgRNA45 and 3, or sgRNA69 and sgRNA36, respectively. The PAM is highlighted in green, the triangle points to the Cas9 cut site; -, deleted base, bold, inserted base. The sequence encoding the TIFY motif is underlined.

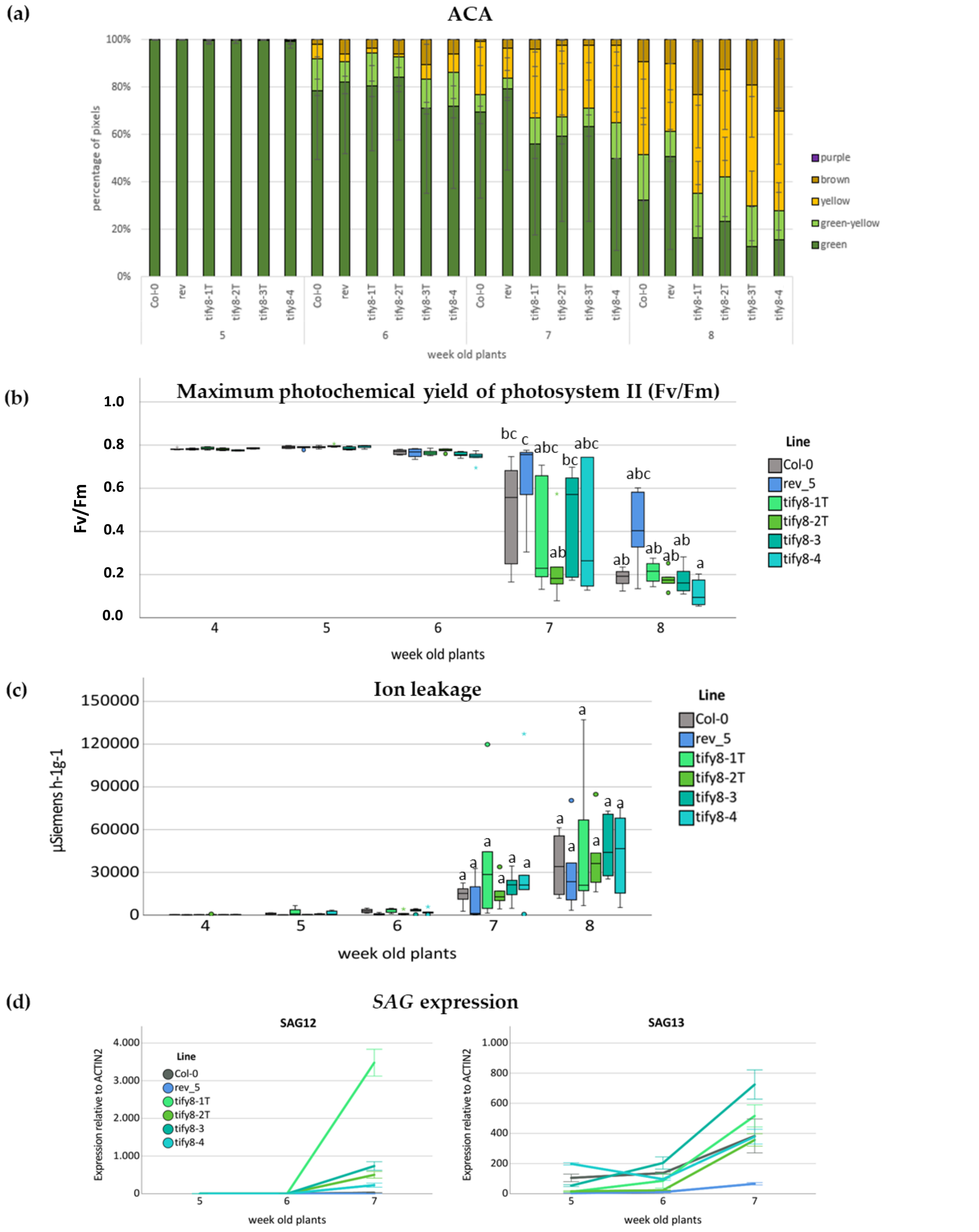

Figure S4: *Senescence parameters of all mutant lines*.
These parameters were analyzed for the senescence phenotyping of *tify8-1T*, *tify8-2T*, *tify8-3*, *tify8-4*, and *rev5* mutants compared to wildtype Col-0 plants. **(a)** Automated Colorimetric Assay (ACA) to categorize the color of individual leaves of at least six plants pixelwise into five groups: green, green-yellow, yellow, brown, and purple. The percentage of each group with respect to total pixel number of all leaves is presented (*n*=6). **(b)** Boxplot of Fv/Fm values measured with PAM for leaves No. 5 of 4- to 7-week-old plants (mean values ±SE, *n*=6-8). **(c)** Boxplot of the decrease in solute retention determined through ion leakage in Leaves No. 4 of 4- to 7-week-old plants (*n*=6-8). One-way ANOVA test was performed, ($p \leq 0.05$). **(d)** Gene Expression of the senescence-associated marker genes *SAG12* and *SAG13* were analyzed by qRT-PCR and normalized to the expression of the *ACTIN2* gene. The expression of both senescence-associated marker genes was analyzed in 5- to 7-week-old plants (mean values ± SD, *n*=3).

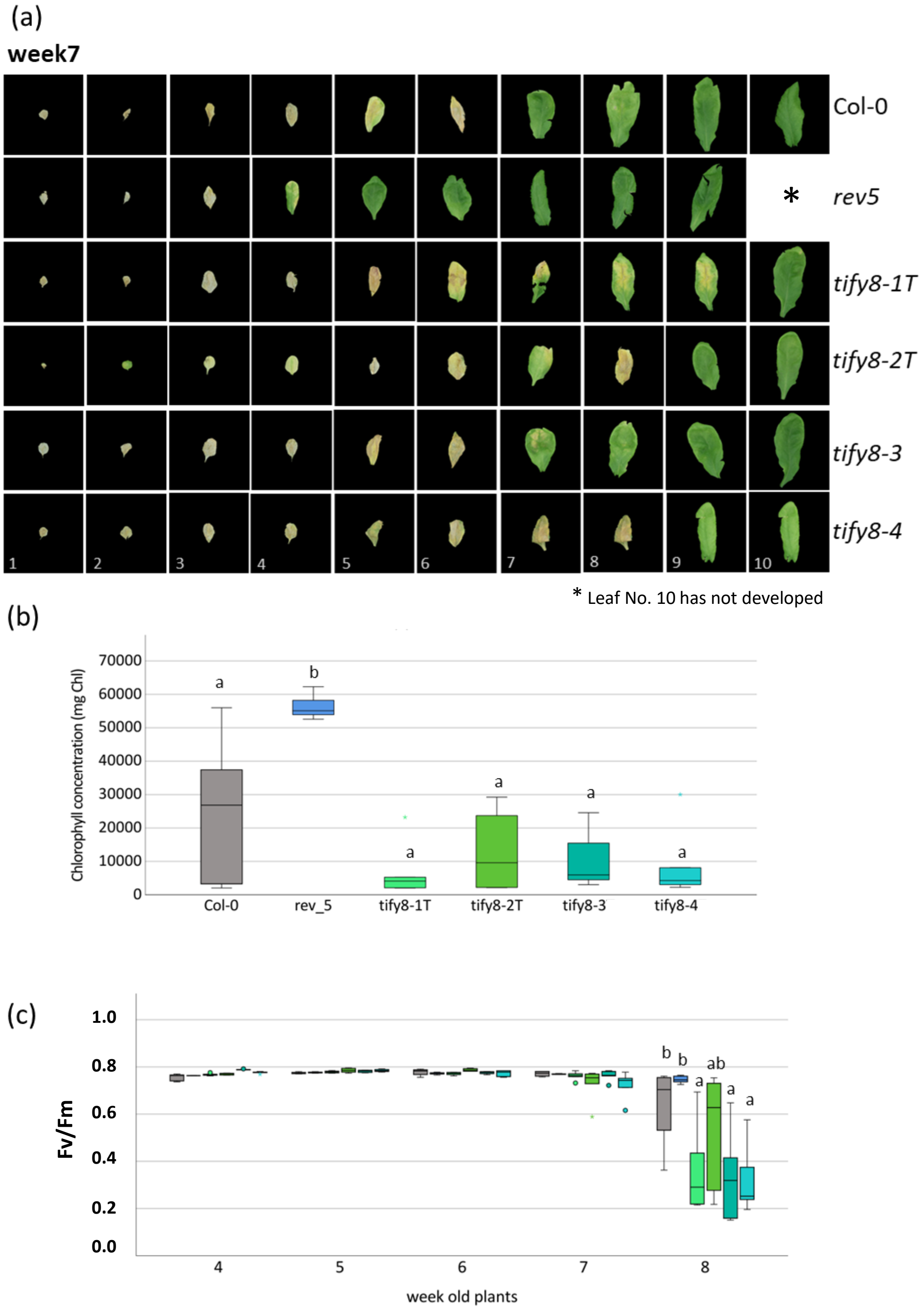

Figure S5*: Phenotypic and photosynthetic parameters.*

These parameters were analyzed for the senescence phenotyping of *tify8-1T, tify8-2T, tify8-3, tify8-4,* and *rev5* mutants compared to wildtype Col-0 plants. **(a)** Phenotypical appearance of leaves at positions 1 to 10 of 7-week-old plants. Boxplots of **(b)** the chlorophyll content per leaf determined for leaves No. 10 of 7-week-old plants (n=6) and **(c)** Fv/Fm values measured by PAM for the identical leaves (No. 10) of 4- to 7-week-old plants (n=6). One-way ANOVA test was performed, ($p \leq 0.05$).

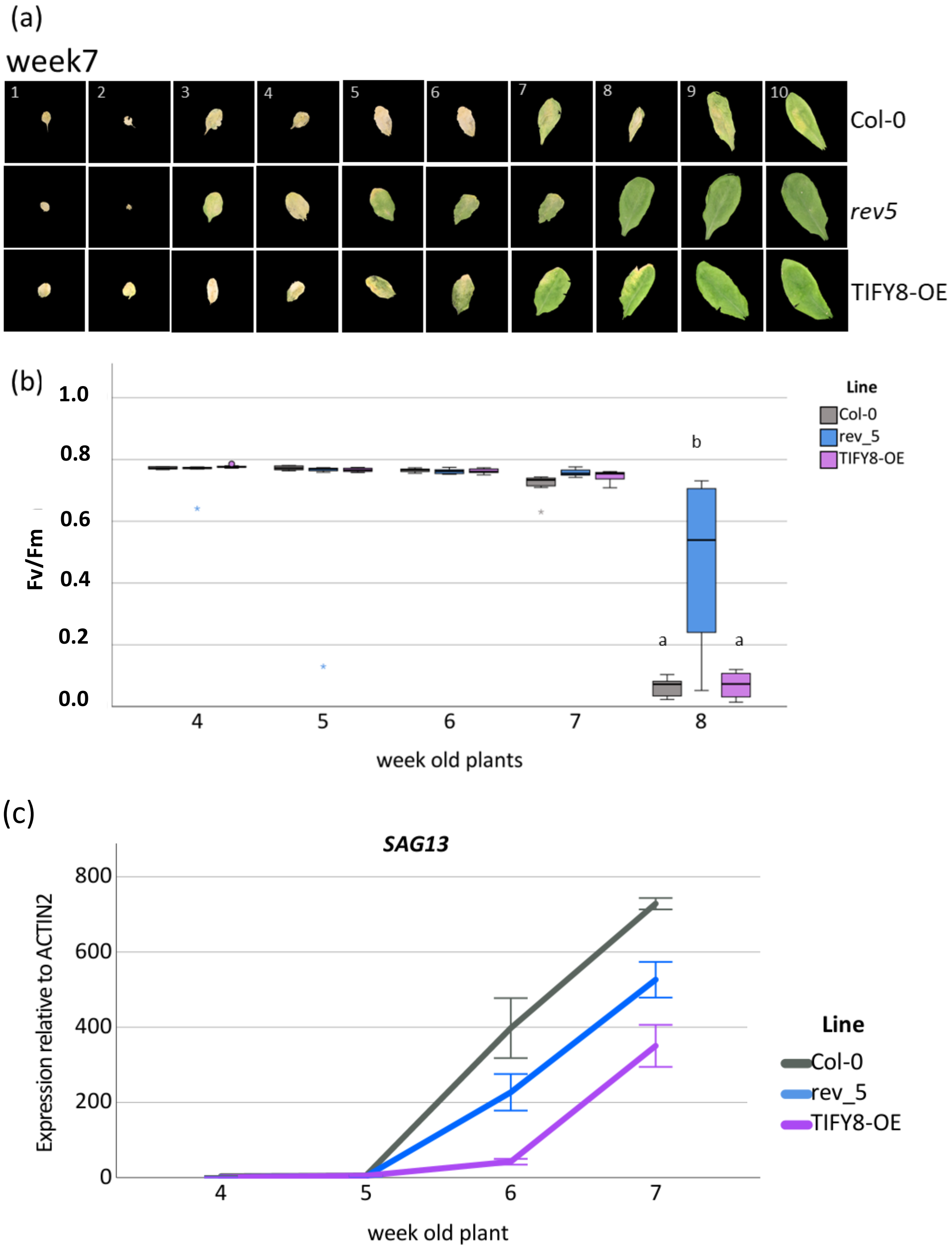

Figure S6: *Senescence parameters.*
These parameters were analyzed for the senescence phenotyping of *TIFY8*-OE and *rev5* mutants compared to wildtype Col-0 plants. **(a)** phenotypical appearance of leaves at positions 1 to 10 of 7-week-old plants, **(b)** Boxplot of Fv/Fm values measured with PAM for the leaves No. 10 of 4 to 7-week-old plants (n=8). One-way ANOVA test was performed, ($p \leq 0.05$). **(c)** Gene expression of the senescence-associated marker gene *SAG13* was analyzed by qRT-PCR and normalized to the expression of the *ACTIN2* gene. The expression was analyzed in 4- to 7-week-old plants in 2 pools of 4 leaves of 4 different plants (mean values ± SD).

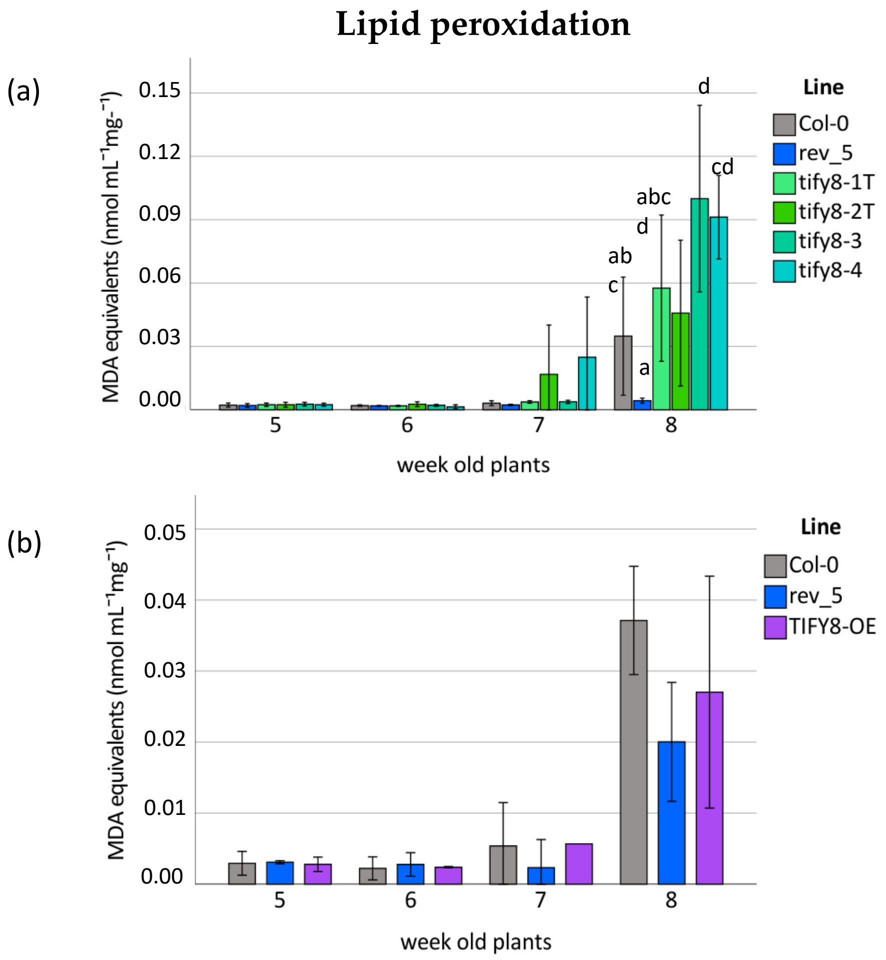

Figure S7: *Lipid peroxidation.*
Lipid peroxidation were determined by the quantification of the MDA concentration in leaves No. 9 of 5- to 7-week-old plants (a) *tify8-1T, tify8-2T, tify8-3, tify8-4*, and *rev5* mutants compared to wildtype Col-0 plants, mean ± SE, *n*=6 , One-way ANOVA test was performed, ($p \le 0.05$) (b) *TIFY8*-OE and *rev5* mutants compared to wildtype Col-0 plants, mean ± SE, (2 pools of 3 leaves No. 9 of 3 different plants each)

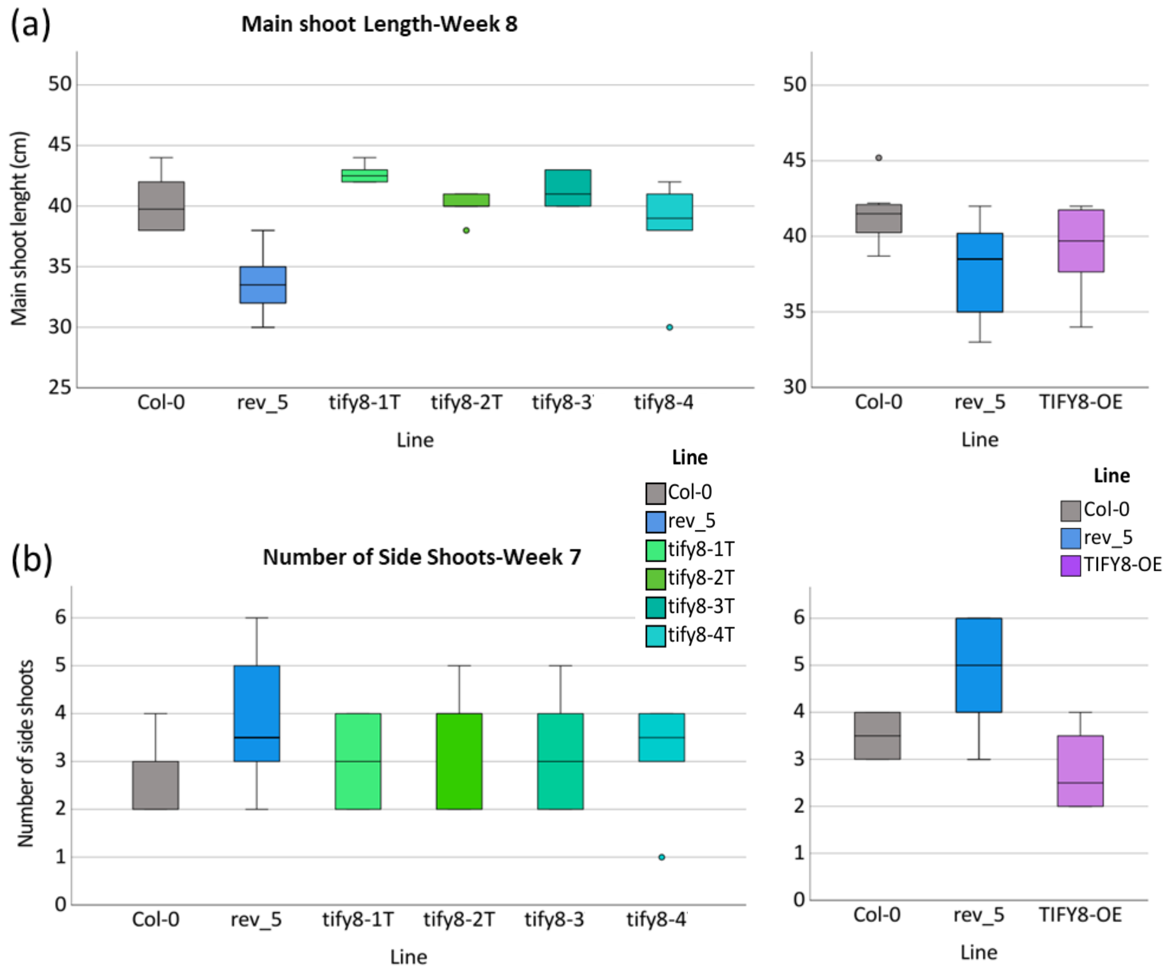

Figure S8: *Phenotypic appearance.*
Boxplots of **(a)** the length of the main shoot measured in 8-week-old plants in all *tify8* mutants as well as in the *TIFY8*-OE and the respective control Col-0 (*n*=6-8). **(b)** the number of side shoots determine in 7-week-old-plants in all the different lines previously mentioned (*n*=6-8)

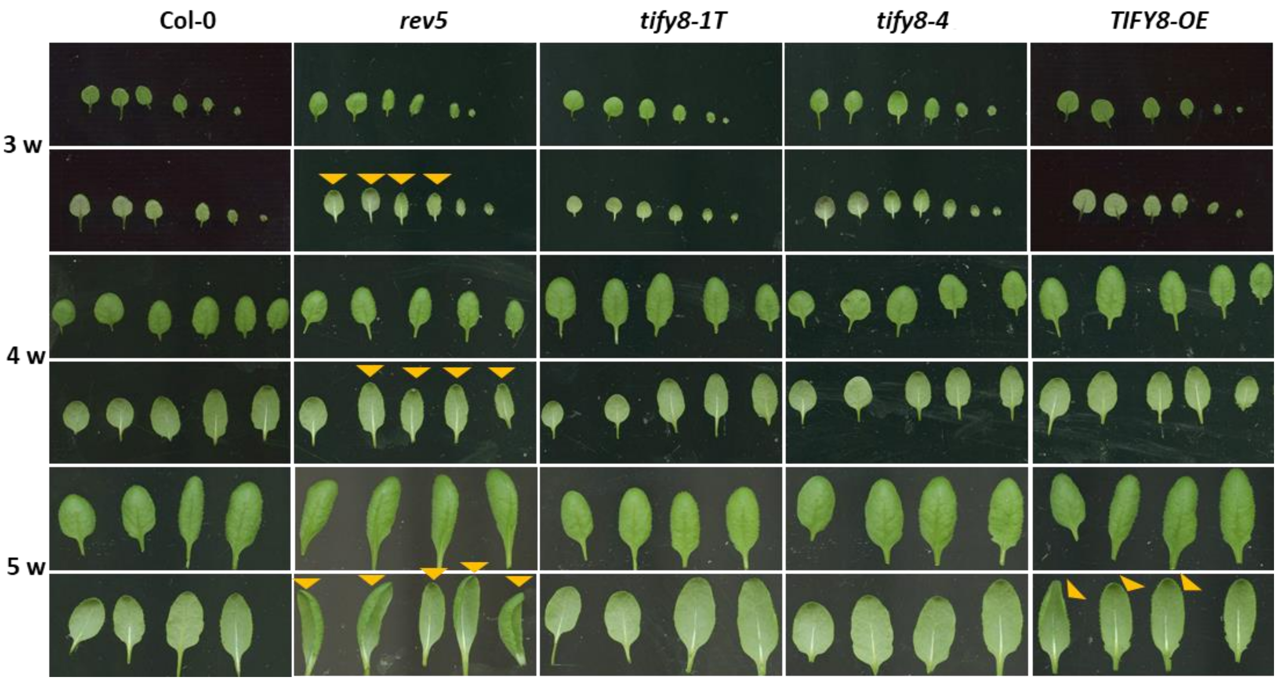

Figure S9: *Leaf phenotype in early stages.*

Leaf development of rosette leaves was documented in early stages of leaf and rosette development. Orange arrow heads point to the downward curled leaves which were typically observed in *rev5* mutant plants.

(a)

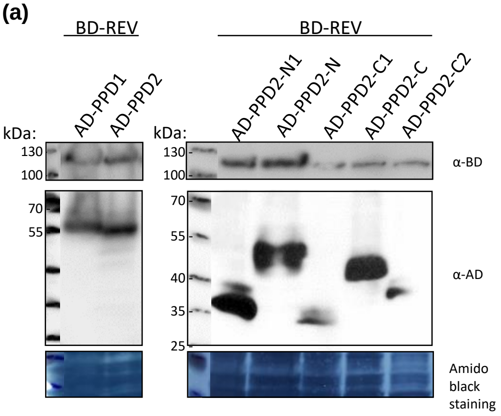

(b)

BD	AD	TDO	DDO/X/AbA	QDO	QDO/X	QDO/X/AbA
REV	PPD1	83%	67%	67%	100%	50%
REV	PPD2	100%	43%	71%	100%	29%
REV	PPD2-N1	0%	0%	0%	0%	0%
REV	PPD2-N	100%	0%	100%	100%	0%
REV	PPD2-C1	0%	0%	0%	0%	0%
REV	PPD2-C	100%	0%	0%	0%	0%
REV	PPD2-C2	0%	0%	0%	0%	0%
REV	empty	0%	0%	0%	0%	0%

100%
83%
71%
67%
50%
43%
29%
0%

Figure S10: *Yeast two-hybrid protein expression in yeast cells and heat map of positive results in independent mating experiments.*
(a) Western blot analyzed with anti-BD and anti-AD antibodies (α) show expression of BD-REV and AD-PPD fusion proteins in diploid yeast cells. The amount of protein loaded onto the gel is indicated by a section of the Amido Black Staining. BD-REV: 114 kDa, AD-PPD1: 58 kDa, AD-PPD2: 58 kDa, AD-PPD2-N1: 37 kDa, AD-PPD2-N: 46 kDa, AD-PPD2-C1: 33 kDa, AD-PPD2-C: 46 kDa, AD-PPD2-C2: 36 kDA. **(b)** Heatmap of positive interaction results of 3 to 7 independent mating experiments in % positive results.

(a)

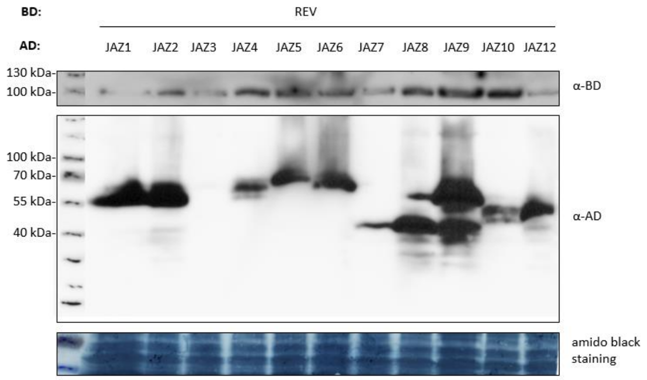

(b)

BD	AD	TDO	DDO/X/AbA	QDO	QDO/X	QDO/X/AbA
REV	JAZ1	100%	100%	100%	100%	100%
REV	JAZ2	100%	25%	100%	75%	0%
REV	JAZ3	75%	50%	50%	75%	25%
REV	JAZ4	100%	50%	100%	100%	25%
REV	JAZ5	100%	0%	100%	100%	0%
REV	JAZ6	100%	0%	50%	0%	0%
REV	JAZ7	0%	0%	0%	0%	0%
REV	JAZ8	100%	0%	50%	25%	0%
REV	JAZ9	100%	0%	100%	100%	0%
REV	JAZ10	100%	0%	75%	75%	0%
REV	JAZ12	100%	0%	0%	0%	0%
REV	empty	0%	0%	0%	0%	0%

100%
75%
50%
25%
0%

Figure S11: *Yeast two-hybrid protein expression in yeast cells and heat map of positive results in independent mating experiments.*
(a) Western blot analyzed with anti-BD and anti-AD antibodies (α) show expression of BD-REV and AD-JAZ fusion proteins in diploid yeast cells. The amount of protein loaded onto the gel is indicated by a section of the Amido Black Staining. BD-REV: 114 kDa, AD-JAZ1: 51 kDa, AD-JAZ2: 51 kDa, AD-JAZ3: 62 kDa, AD-JAZ4: 58 kDa, AD-JAZ5: 54 kDa, AD-JAZ6: 54 kDa, AD-JAZ7: 41 kDa, AD-JAZ8: 39 kDa, AD-JAZ9: 53 kDa, AD-JAZ10: 46 kDa, AD-JAZ12: 44 kDa. **(b)** Heatmap of positive interaction results of 3 to7 independent mating experiments in % positive results.

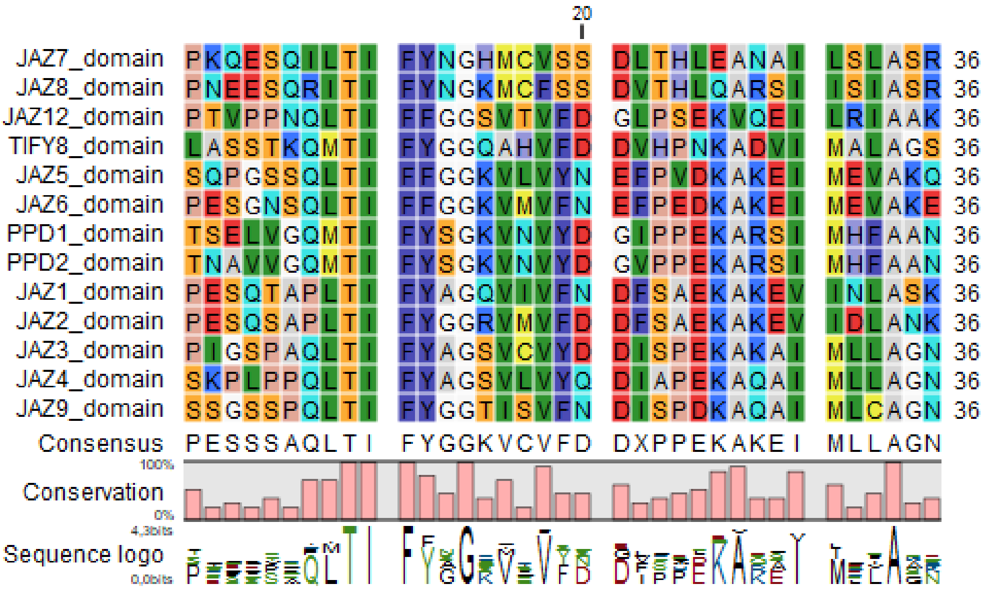

Figure S12: *Alignment of all TIFY/JAZ domains of the class two TIFY proteins.*
